# Crossed Renal Ectopia without Fusion: An Uncommon Cause of Abdominal Mass

**DOI:** 10.1155/2015/679342

**Published:** 2015-07-28

**Authors:** Ana Ratola, Maria Miguel Almiro, Rita Lacerda Vidal, Nuno Neves, Adelaide Bicho, Sofia Figueiredo

**Affiliations:** ^1^Pediatrics Department, Baixo Vouga Hospital Center, Avenida Artur Ravara, 3814-501 Aveiro, Portugal; ^2^Radiology Department, Baixo Vouga Hospital Center, Avenida Artur Ravara, 3814-501 Aveiro, Portugal

## Abstract

Crossed renal ectopia is a rare congenital anomaly usually associated with fused kidneys (90%). Most cases are asymptomatic and remain undiagnosed. We report an unusual case of nonfused crossed renal ectopia. The 11-year-old adolescent female patient was admitted with abdominal pain, anorexia, weight loss, and periumbilical mass. Although the initial clinical suspicion was a tumoral lesion, abdominal ultrasound and magnetic resonance examination revealed crossed renal ectopia without fusion. The renal ectopy was incidentally diagnosed, as described in 20 to 30% of cases. In this case, the associated nonspecific symptoms were a coincidence.

## 1. Introduction

Crossed renal ectopia (CRE) is a rare congenital anomaly consisting of the transposition of a kidney to the opposite side. Nevertheless, the ectopic kidney's ureter crosses the midline to insert in its normal position in the bladder [1]. Ninety percent of the crossed ectopic kidneys are fused [2–4].

The majority of pediatric patients are asymptomatic, but they often have nonspecific or unrelated symptoms such as abdominal or flank pain, palpable mass, hematuria, or dysuria. In some reported cases, the patient develops additional symptoms arising from complications such as infection, renal calculi, or urinary obstruction [5]. Accordingly, most cases remain unnoticed whereas 20 to 30% of the cases are only incidentally detected [1–4].

Here, we report the case of crossed nonfused renal ectopia incidentally diagnosed.

Given that the adolescent had an abdominal mass and pain, it was therefore necessary to discard other diagnoses first.

## 2. Case Report

An 11-year-old adolescent female was admitted to the emergency department with lower abdominal pain for 2 months associated with anorexia and weight loss of about 2 kg in the last month (approximately 4.3% of body weight loss). This was the first time these complaints required medical assistance. The patient had a healthy appearance, but the physical examination revealed a periumbilical, well-defined, and painless mass (9 × 6 cm) and a stool-filled dilated descending colon. The weight, height, and body mass index were, respectively, at the 81st, 83rd, and 78th percentiles on the CDC pediatric growth charts (see Supplementary Material available online at http://dx.doi.org/10.1155/2015/679342) [6]. Breast and pubic hair development were Tanner stage 4 [7]. Blood pressure (BP) measures were below the 50th percentile on BP tables for children and adolescents, by height, sex, and age [8].

Constipation was the only condition reported in the personal medical history. The prenatal ultrasounds scans did not detect any structural abnormalities and were described as normal and no urinary tract infections were diagnosed in the past. No relevant family background was found.

Based on the clinical data (abdominal mass, anorexia, and weight loss), an abdominal tumoral lesion was initially suspected; namely, the most common in this age was lymphoproliferative disease (lymphoma), germ cell tumor (teratoma, germinoma), or sarcoma. However, an abdominal ultrasound was performed and revealed crossed ectopic left kidney not fused to the right kidney. Both kidneys showed normal renal parenchymal thickness, echogenicity, and parenchyma-sinus differentiation. The left ectopic kidney was located in the right hemiabdomen with the hilum anteriorly faced.

The discharge planning took into account the good health appearance and the fact that the adolescent's growth had not crossed the percentile lines (see Supplementary Material). It was recommended to increase fluid and dietary fiber intake and a laxative treatment was prescribed. The patient was reevaluated 3 weeks later in pediatric nephrology consultation. At this time, she already had recovered the previous weight (see Supplementary Material) and the symptoms of constipation had improved.

**Figure 1:**
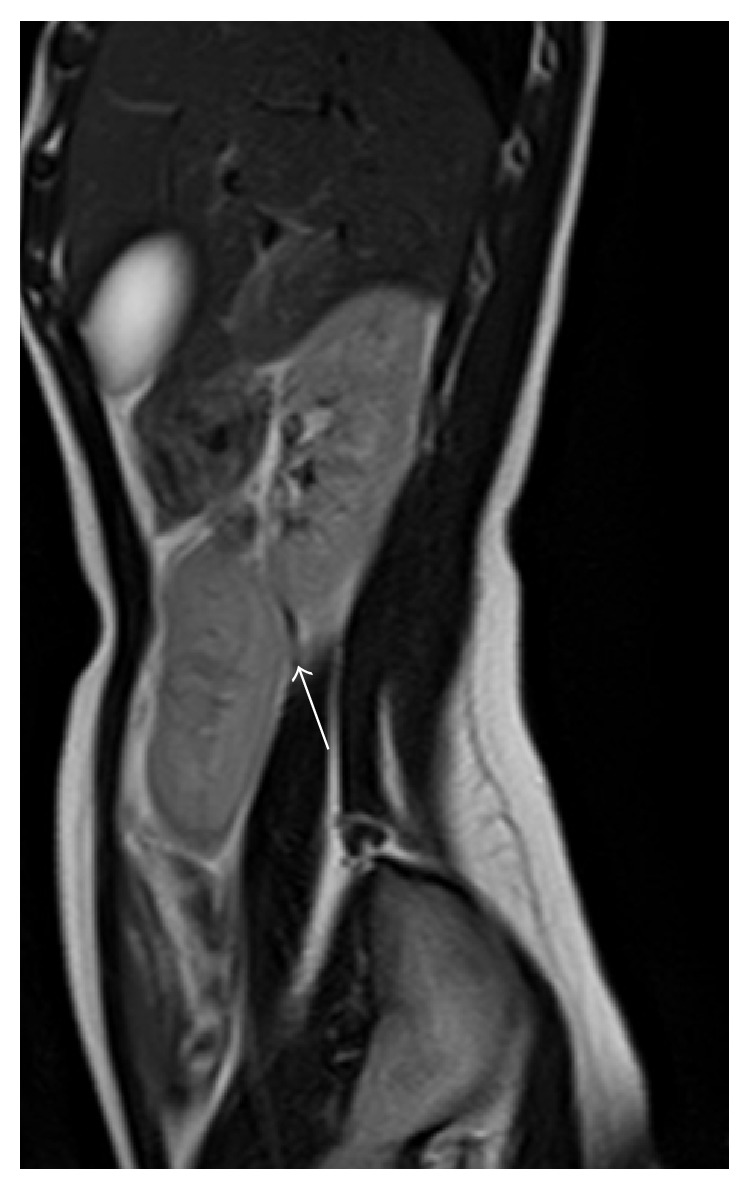
11-year-old adolescent female with left-to-right crossed renal ectopia. Sagittal RM scan, showing a clear plane of separation between the two kidneys (white arrow) and each kidney, having its own Gerota's fascia.

**Figure 2:**
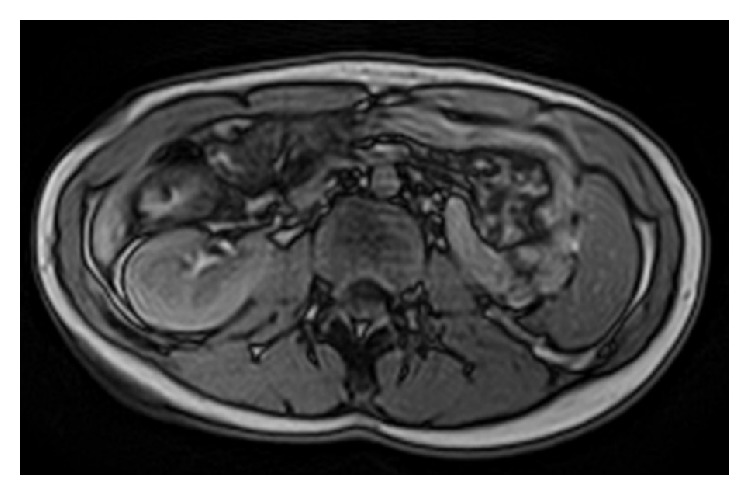
11-year-old adolescent female with left-to-right crossed renal ectopia. Axial RM scan demonstrates two normal enhancing kidneys on the right.

To complete the renal ectopia investigation, a magnetic resonance imaging and DMSA renal scintigraphy were performed. The magnetic resonance imaging showed the left kidney on the right side, slightly below and anterior to the other kidney (Figures 1 and 2). It also revealed a malrotation of the ectopic kidney with the renal pelvis anteriorly oriented and an apparent left ureteropelvic duplication without dilatation of the excretory system. DMSA renal scintigraphy revealed left renal crossed ectopy, with preserved differential function (46.2% in the left kidney) and without renal scarring. One year follow-up of the patient revealed no further symptoms or no additional complications.

## 3. Discussion

Renal ectopy and fusion are common congenital anomalies of the kidney and urinary tract, and they result from the disruption of the normal embryologic migration of the kidneys [5]. Renal ectopy occurs when the kidney does not ascend normally to the retroperitoneal renal fossa (level of the second lumbar vertebra) [4, 5, 9]. Simple congenital ectopy refers to a kidney that lies on the correct side of the body but in an abnormal position. When kidneys cross the midline the condition is known as CRE. This can occur with or without fusion to the contralateral kidney [5].

CRE can be anatomically differentiated into four groups: (1) CRE with fusion (the majority of cases, 90%); (2) CRE without fusion (uncommon); (3) solitary CRE (very rare); and (4) unfused bilateral CRE (also very rare) [1–3]. In the first two groups, the ectopic kidney is usually located below the orthotopic kidney. Malrotation of the crossed ectopic kidney is the predominant form. CRE is more frequent in males (M/F = 1.4/1) and is two to three times more common on the right than on the left side [1].

Our patient had the uncommon CRE without fusion identified after investigation of nonspecific symptoms (abdominal pain and palpable mass). The pain was probably associated with constipation. Although weight loss and anorexia were unrelated to the anomaly, these features made the ectopic kidney palpable, facilitating the diagnosis. The renal ectopy was therefore incidentally diagnosed as it occurs in 20 to 30% of the cases [2].

A high incidence of other urological abnormalities has been associated with renal ectopy [1–5]. Vesicoureteral reflux is the most common and occurs in 20% of the CRE cases [5, 9]. Other genitourinary abnormalities include megaureter, hypospadias, cryptorchism, urethral valve and cystic dysplasia, and unilateral agenesis of fallopian tubes and ovaries [1, 5, 10]. Our patient had an apparent left ureteropelvic duplication without dilatation of the bilateral excretory system. No other anomalies were found.

Ectopic kidney can also be associated with other nonrenal anomalies (adrenal, cardiopulmonary, gastrointestinal, and skeletal abnormalities) and genetic syndromes [1, 4, 5, 10].

Moreover, ectopic kidneys are likely to be associated with urological complications, such as urinary infections, renal calculi, and ureteropelvic junction obstruction, due to their frequent abnormal shape, malrotation, and aberrant vasculature [1, 5]. When an ectopic kidney is detected, associated renal and urinary anomalies and structural extrarenal malformations should be evaluated [10].

## 4. Conclusion

CRE without fusion is a rare condition that can be diagnosed when other diseases are being investigated, as in this case. Treatment is only indicated for the complications.

Patients may need a follow-up and should be examined to check for potential complications.

## Supplementary Material

In this section, we present the CDC growth charts (weight and BMI) for the patient in question, at the time of the admission to the emergency department and in the follow-up three weeks later.
