# Supplementary material for: Crossed Renal Ectopia without Fusion: An Uncommon Cause of Abdominal Mass
Source: Case Rep Nephrol. 2015 Jul 28;2015:679342. doi: 10.1155/2015/679342 (PMC4531181; doi:10.1155/2015/679342)
Supplement: Supplementary file 1 — In this section, we present the CDC growth charts (weight and BMI) for the patient in question, at the time of the admission to the emergency department and in the follow-up three weeks later. [file 679342.f1.pdf]

CDC Growth Charts: United States

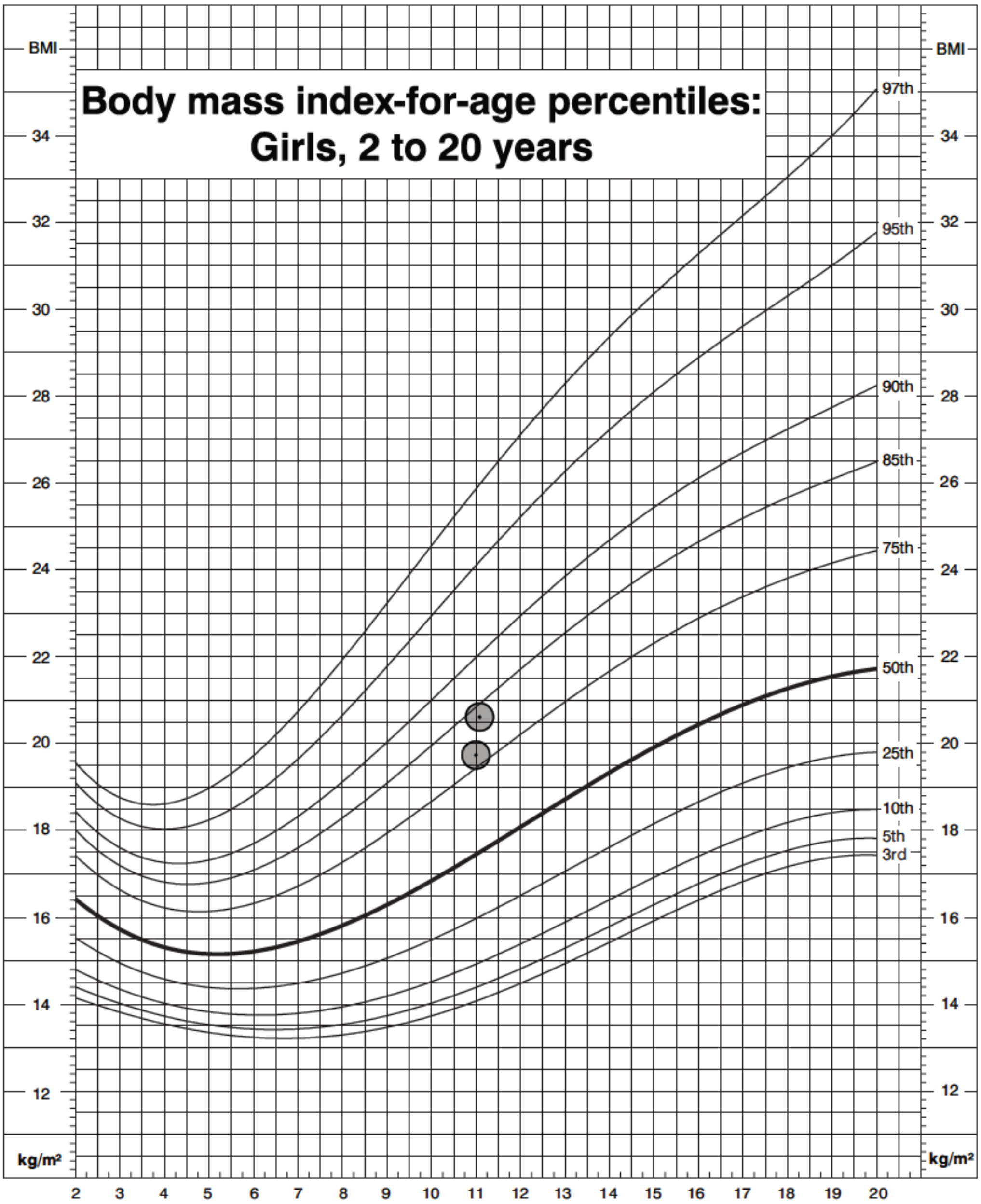

Published May 30, 2000.
SOURCE: Developed by the National Center for Health Statistics in collaboration with the National Center for Chronic Disease Prevention and Health Promotion (2000).

SAFER · HEALTHIER · PEOPLE™

CDC Growth Charts: United States

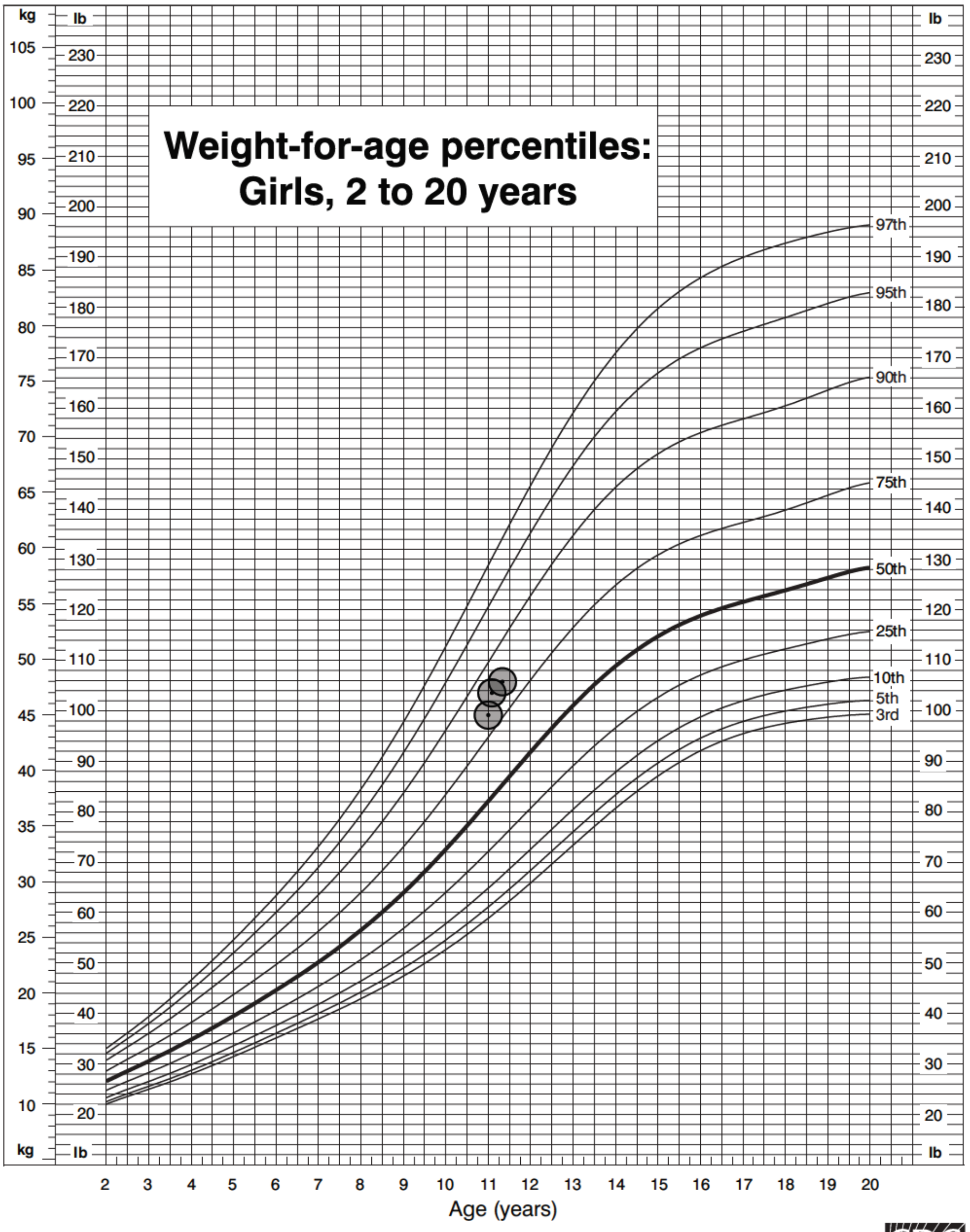

Published May 30, 2000.
SOURCE: Developed by the National Center for Health Statistics in collaboration with the National Center for Chronic Disease Prevention and Health Promotion (2000).

SAFER · HEALTHIER · PEOPLE™
